# Crude extract of *Ficus deltoidea* Jack (FD) as a natural biological therapy

**DOI:** 10.37349/etat.2023.00123

**Published:** 2023-02-28

**Authors:** Mahmoud Dogara Abdulrahman

**Affiliations:** Biology Education Department, Faculty of Education, Tishk International University, Erbil 44001, Kurdistan Region, Iraq; NGO Praeventio, Estonia

**Keywords:** Anticancer, medicinal plants, preclinical

## Abstract

**Aim:**

This study shows how important it is to coordinate research on *Ficus deltoidea* Jack (FD) so that results from different sources can be compared directly and a scientific conclusion can be made.

**Methods:**

The author looked for research papers on *Ficus* (*F.*) *deltoidea* on Google Scholar, Science Direct, Google.com, Wiley, PubMed, Hindawi, Springer, and other related databases. This analysis excludes data that cannot be trusted, thesis papers, and review articles about *F. deltoidea*.

**Results:**

In traditional medicine, the plant’s leaves and syconia are used to cure a wide variety of ailments, including itchiness, diarrhoea, cancer, sexual dysfunction, age-related issues, malaria, cancer, anxiety, pain, constipation, fever, diabetes, tooth pain, and tooth decay. *In vitro* and *in vivo* studies showed the effectiveness of the leaves against cancer cell lines.

**Conclusions:**

Based on the existing research on the health benefits of FD, it is critical to focus on its more active constituents and their identification, determination, further development, and, most importantly, standardization of the leaves for the management and treatment of cancer and its related cases. More research is needed before it can be considered a promising herbal source of novel medication candidates for treating various disorders.

## Introduction

Nature provides numerous plants that serve as the primary source of traditional medicines that can treat a wide range of illnesses [1]. Humans have used medicinal plants for thousands of years as a source of antimicrobial, antifungal, and anticancer agents, and for many other uses [2]. People have been very interested in biological products for a long time. Discovering new compounds with potential future applications is one of the main motivations for researching these priceless by-products [3]. Plant-based remedies for health issues have been on the rise recently. The need for new drugs derived from numerous species of medicinal plants is continually growing today [1]. Investigating potent natural compounds from plants with high biological activity is still ongoing. *Ficus deltoidea* Jack (FD) is one of the most well-known and widely appreciated plants. Many studies have been published on the plant’s biological properties. The current literature [4, 5] on its potential for managing and treating diseases, especially cancer, needs to be reviewed, analysed, and brought up to date. This study combines the scattered data on the biological impacts of *Ficus* (*F.*) *deltoidea* and synthesizes the data into a cohesive whole, paving the way for a more thorough understanding of the plant and for a clearer guidance on how to make the best use of its components.

## Materials and methods

**Figure 1.**
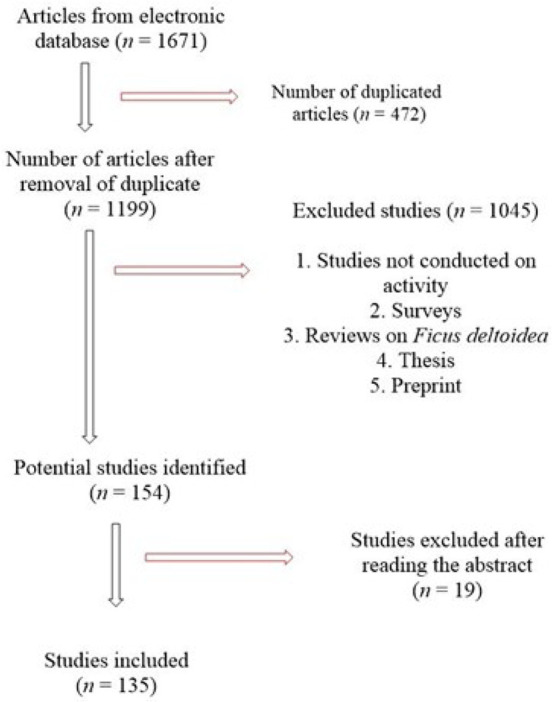
Methodological flow diagram showing inclusion and exclusion criteria

**Inclusion criteria:** A comprehensive search of online resources like Web of Science, Taylor and Francis’ Science Direct, Google Scholar, Scopus, Springer Link, PubMed Central, SciELO, and Elsevier databases. Keywords such as *Ficus deltoidea*, *Ficus deltoidea* Jack, *F. deltoidea* in combination with antimicrobial, antioxidants, anticancer, anti-inflammation, anti-inflammatory, and other related and relevant phrases were used to search in the above databases. No time constraints were imposed, and all relevant databases were considered (Figure 1). **Exclusion criteria**: Only published research articles were considered; reviews, thesis abstracts, and unpublished papers were excluded (Figure 1).

## Results

### Origin, distribution and taxonomic distribution of *F. deltoidea*

About 1,000 species from all over the tropics and subtropics make up the genus *F.* [6]. *F. deltoidea* is a species of shrub that is indigenous to Southeast Asia. It is known in Malay as the mistletoe fig or Mas cotek [7]. It is also called Sempit-sempit or agolaran by southern Malays [8]. In Central Africa, people call it Kangkaliban, but people call it Tabat Barito in Indonesia. *F. deltoidea* is indigenous to several Southeast Asian countries and may be seen growing widely throughout the region [9]. However, this plant can also be found in Africa [10]. This type of plant is usually found in Malaysia, Indonesia, and the southern Philippines, all of which are in Southeast Asia [8]. FD may be found in tropical and subtropical regions and comes in several different types [11]. FD is a natural shrub from the Moraceae family [8]. The form of the *F. deltoidea* leaves led to the separation of this species into two subspecies: *F. deltoidea subsp. motleyana* and *F. deltoidea subsp. deltoidea* [12]. There are two kinds of *F. deltoidea* plants: male and female. The difference between a male plant and a female plant is that the male plant has longer leaves, while the female plant has big, round, and long leaves [13]. The evergreen little tree or shrub can grow to 7–10 meters in its natural habitat [8]. The local people cultivate FD as a houseplant for aesthetic and medical advantages [14].

### Traditional uses

The plant is well-known among the Malay people and is utilised in treating diabetes, headaches, sore throats, and colds [7]. Traditional medicine uses various sections of the plant to cure various conditions [15]. In traditional medicine, hyperlipidemia, hypertension, and diabetes are all treated with *F. deltoidea* [7, 12]. This plant plays a significant role in traditional medicine, with its fruit used to treat a wide range of ailments, from headaches and toothaches to wounds (roots and leaves) [8, 15]. The consumption of fruit is a common method for alleviating pain associated with toothache and migraine, root and leaf remedies for cuts and scrapes [8]. After giving birth, women drank a decoction of the leaves to help tighten the uterine and vaginal muscles [8, 15]. It has been theorized that drinking a concoction made from the leaves can increase blood flow, have aphrodisiac effects, and even can fight diabetes [13]. Traditional uses for the extract include treating wounds, rheumatism, and ulcers; it is also effective as an antidiabetic medicine and a tonic for usage after giving birth [16].

### Antioxidants activity

**Figure 2.**
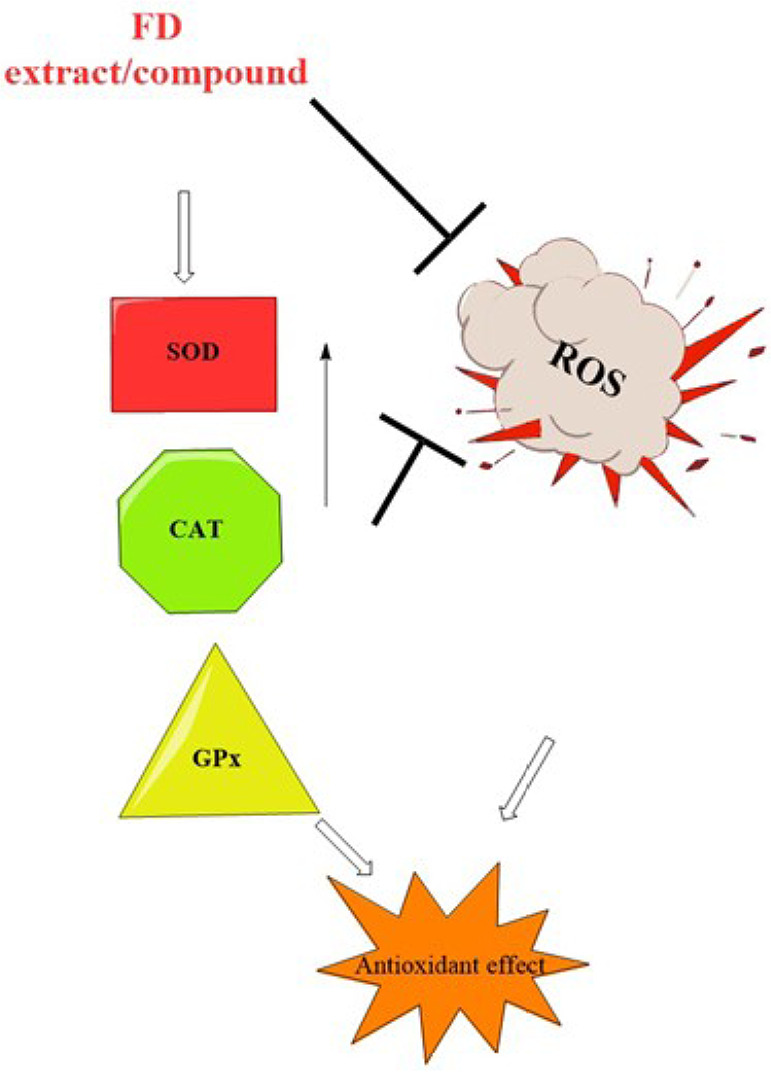
Possible mechanism of antioxidant activities of FD; SOD, CAT, GPx (antioxidant enzymes)

An antioxidant defence system is in place to counteract the oxidative stress caused by the body’s normal physiological process of radical and reactive oxygen species (ROS) formation [17]. ROS are made when oxidative stress and the antioxidative defence system are out of balance. ROS can damage lipids, carbohydrates, proteins, and DNA, leading to many diseases [17]. Because of antioxidants’ ability to protect the body from harmful free radicals and ROS, many chronic diseases can be avoided and even reversed. Different parts of *F. deltoidea* were evaluated for antioxidant potential (Table 1). Antioxidant activities of *F. deltoidea* have been documented in several investigations, but the portion of the plant utilized in the vast majority of the studies was the leaf [18]. The removal of ROS by the hydrolysed protein fractions was superior to that by the unhydrolyzed protein fractions [19]. Based on a one-way analysis of variance, only the protein hydrolysates of 30 and 100 kDa indicated significant differences in radical scavenging capacities [19]. Methanolic leaf extract had the highest antioxidants for ferric reducing antioxidant power (FRAP) (6–9 mmol Fe^2+^/g), 2,2’-azino-bis (3-ethylbenzothiazoline-6-sulfonic acid) (ABTS) (2–3 mmol TE/g), and 2,2-diphenyl-1-picrylhydrazyl (DPPH) (EC50:200–410 μg/mL) [12]. The methanol extract of *F. deltoidea* was the most effective at scavenging free radicals at 400 μg/mL (85.41%). Lacklustre radical scavenging activity was observed in butanol extract [1]. The findings of this study revealed that solvent extracts play a critical role in demonstrating biological activities. It was discovered that antioxidant and total phenolic content (TPC) depend on the polarity of the solvent in the case of antioxidant activity [1]. Eighty-five per cent of the antioxidant activity of the FD extract was attributed to flavan-3-ol monomers and proanthocyanidins [20]. Based on these results, it is plausible that the leaves of *F. deltoidea* could be employed as a natural antioxidant. These enzymes’ activity and protein levels were elevated after exposure to *F. deltoidea* extract, suggesting that this compound may be responsible for reducing ROS production by acting on these enzymes. As a result, the extract directly scavenges ROS and indirectly stimulates the production of antioxidant enzymes (Figure 2). The main antioxidant defence system consists of antioxidant enzymes like superoxide dismutase (SOD), catalase (CAT), and glutathione peroxidase (GPx). Anti-ageing is partially achieved by SOD’s ability to scavenge the superoxide anion formed in the early stages of oxidative stress. With the help of SOD, superoxide can be converted into harmless hydrogen peroxide and dioxygen. To quickly catalyse the decomposition of hydrogen peroxide, cells frequently use CAT and GPx (Figure 2).

**Table 1.** Antioxidant activities of FD

**Serial number (S/N)**	**Methods**	**Solvent**	**Plant parts**	**Concentrations**	**Major findings**	**Reference**
1	DPPH, FRAP	Methanol, chloroform, ethyl acetate and butanol	Leaves	25–400 μg/Ml	Methanol extract has the strongest scavenging activity. The radical scavenging effect of the methanol extract appeared to be comparable to that of ascorbic acid at a concentration of 100 μg/mL, while the reducing power of all extracts was concentration dependent	[1]
2	DPPH	Aqueous	Leaves and fruits	250, 125, 62.5, and 31.3 μg/mL	The leaves and fruits of var deltoidea demonstrated the maximum radical scavenging activity (82.04 and 71.43 per cent, respectively) in the DPPH experiment	[21]
3	DPPH, FRAP	Methanol	Leaves	100 μL	DPPH radical scavenging activity revealed a half maximal inhibitory concentration (IC_50_) value of 66.81–288.04 μg/mL and reduced power activity at 0.02–0.24 μg/mL	[22]
4	DPPH	Methanol	Leaves	100, 50, 25, 12.5, and 6.25 ppm	The extract of the leaves has antioxidant activity at IC_50_ = 72.47 μg/mL	[23]
5	DPPH		Leaves		In a DPPH experiment, the aqueous extract of female *F. deltoidea* leaves has an IC_50_ value of 29 μg/mL, while the aqueous extract of male leaves has an IC_50_ value of 40.1 μg/mL	[24]
6	DPPH, FRAP	Ethanol, aqueous	Fruits		Regarding DPPH radical scavenging activities, the extract’s IC_50_ values ranged from 13.5 to 79.3 μg/mL. The extracts’ ability to reduce Fe^3+^ to Fe^2+^ revealed that almost all of them have significant reducing power. The FRAP values of the extracts ranged from 1.1 to 9.72 mmol/g, respectively	[25]
7	DPPH, FRAP	Hot and cold aqueous	Leaves	DPPH 40 μL and FRAP 200 μL	In terms of radical scavenging assay, the cold aqueous extract has the highest percentage of inhibition at 46.77, while FRAP has a similar percentage at 93.69	[26]
8	DPPH	Hot aqueous	Leaves	100 μL	The amount of total phenolic and radical scavenging activity has a positive linear relationship (R^2^ = 0.65–0.76). According to the research, the *F. deltoidea* leaf can provide phenolic antioxidants	[27]
9	DPPH, lipid peroxidation, scavenging, FRAP, total antioxidant capacity assays	Methanol	Leaves	100 mL	All the methods exhibited good activity, with DPPH presenting the IC_50_ value at 14.1 μg/mL	[28]
10	DPPH, FRAP	Methanol, ethanol	Leaves	40 μL	While ethanol extraction had the highest total antioxidant activity (DPPH) (4.48 mg TE/g FW), methanol extraction had the highest total antioxidant activity (FRAP) (2.43 mg TE/g FW)	[29]
11	DPPH	Aqueous	Leaves	1–100 μg/mL	The results showed that the extract was most effective at getting rid of free radicals with an IC_50_ of 0.039 mg/mL	[30]
12	DPPH	70% methanol	Leaves	5 μL	Only about 30% of DPPH could be inhibited at the highest possible dose of the plant extract	[10]
13	FRAP, DPPH	Hexane, ethyl acetate, methanol, water	Leaves	10 μL and 0–2,000 μg/mL	At the activities of FRAP (6–9 mmol Fe^2+/^g), ABTS (2.0–3.0 mmol TE/g), and DPPH (EC_50_: 200–410 μg/mL), methanolic leaf extract had the highest antioxidants	[12]
14	DPPH	Methanol, ethanol, aqueous	Leaves	1 and 100 μg/mL	The ethanolic extract had the lowest IC_50_ value, followed by the methanolic extract (22 μg/mL) and the aqueous extract (23 μg/mL) on the graph of percentage inhibition against sample concentration	[31]
15	DPPH	Water and ethyl acetate	Leaves	100 μL	Antioxidant potency is measured by the darkening of the reaction mixture in the DPPH assay. Because both types inhibited at least half of the radicals, they were considered equal	[7]
16	DPPH	Methanol	Leaves and stems	100, 50, 25, 12.5, and 6.25 μg/mL, respectively	Compared with stem extract, the IC_50_ of leaf extract exhibits a considerable antioxidant activity (34 and 39 μg/mL extract) based on the radical scavenging activity	[32]
17			Hydrolysed protein		The hydrolysed protein fractions were shown to be more effective at removing ROS than the un-hydrolysed fractions. Only the protein hydrolysates of 30 and 100 kDa revealed significant variations in radical scavenging capabilities based on a one-way analysis of variance	[19]
18	DPPH	Hexane, chloroform and methanol	Fruits	0.75–5.0 μg/mL	The 250 μg/mL of methanol extract and 125 μg/mL of chloroform extract were both able to get rid of more than 50% of free radicals. All extracts were very good at fighting free radicals	[33]
19		Aqueous	Leaves	500 mg/kg	In addition to reducing the amount of malondialdehyde (MDA) in the rats’ organs, the *F. deltoidea* leaf extract also increased glutathione (GSH) and CAT activity while decreasing total cholesterol (TC) levels in their blood. Only the rats’ hearts and kidneys were shown to have increased GSH activity	[34]
20	DPPH	n-hexane, ethyl acetate, methanol	Leaves	7.81–1,000 μg/mL	According to a new study, the IC_50_ value for DPPH radical scavenging activity was 129.27 μg/mL for the methanol extract of *F. deltoidea* leaves as a viable natural antioxidant for medicinal usage	[35]
21					The *F. deltoidea* extract may be useful in anti-photoaging cosmetics because it protects against ultraviolet radiation b (UVB)-induced skin damage	[36]
22	DPPH	Aqueous	Leaves and fruits	250, 125, 62.5, and 31.3 μg/mL	Increases in extract concentration from 31.3 μg/mL to 250 μg/mL increase inhibition	[37]
23		Methanol	Leaves and stems	50 μL	Crushed leaf and stem extracts from female and male *F. deltoidea* plants showed considerable antioxidant activity in the DPPH assay. After the female stem and leaf were extracted, the maximum antioxidant activity was found to be in the female leaf extract (fraction 51), followed by the female leaf extract (fraction 8), and the male leaf extract (fraction 35)	[38]
24	DPPH	Hot aqueous	Fruits	25–1,000 μg/mL	The extracts and fractions found the maximum antioxidant activity and phenolic content, with a total of 121.62 mg/g extracts	[18]
25	DPPH		Leaves		The IC_50_ of the isolated compounds is 92.5 μM for vitexin and 115.4 μM for isovitexin	[39]
26	DPPH	Methanol	Leaves	20 μL	All species were recorded to be significantly active	[40]
27	DPPH, superoxide anion scavenging activity	Methanol	Leaves		Divergent radical scavenging activities (*P* < 0.05) were seen between alcoholic and aqueous extracts of different plant varieties. Both extract types showed significant antioxidant activity in DPPH and superoxide anion scavenging models	[17]
28	DPPH	Methanol	Fresh leaves, senescent leaves, unripe fruits, ripe fruits and stems	100, 50, 25, 12.5, and 6.25 ppm	In terms of antioxidant activity, senescent leaves at 34.1 had the greatest IC_50_ values, followed by fresh leaves at 34.4, matured fruits at 39.4, unripe fruits at 50.2, and stems at 126.1 ppm, respectively	[41]
29		Aqueous			The antioxidant activity of *F. deltoidea* extract was determined by HPLC with online antioxidant analysis. This showed that the flavan-3-ol monomers and proanthocyanidins accounted for 85% of the overall antioxidant activity of the aqueous *F. deltoidea* infusion	[20]

### Anti-inflammation activity

**Figure 3.**
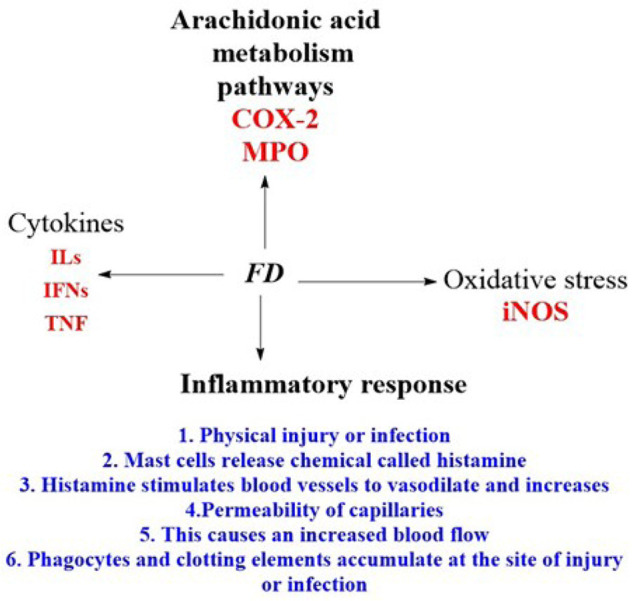
Anti-inflammatory mechanisms of action of *F. deltoidea* crude extract or pure compounds. MPO: myeloperoxidase; IFNs: interferons; iNOS: inducible NOS

The process through which the body reacts to cellular damage is called inflammation [42]. It is a chain of events that can be set off by various stimuli, resulting in a predictable behavioural signature [42]. Results showed that FD aqueous extract (FDA) had significant anti-inflammatory effects in all assays at (*P* < 0.05) [11], and the paw oedema and formalin tests showed dose-response effects. In conclusion, the *F. deltoidea* leaf can reduce short-term and long-term inflammation and pain-related inflammation [11]. The findings, therefore, demonstrated the presence of pharmacologically active compounds with antinociceptive activity in the aqueous extract of *F. deltoidea* leaves [15]. Because of this, it is frequently applied in medicine to treat ailments that cause pain [15]. The fact that the FDA suppressed carrageenan-induced rat paw oedema for 5 h beginning 0.5 h after administration of the phlogistic drug implies that the extract’s mode of action entailed suppressing the cyclooxygenase (COX)-dependent response [42]. The lipopolysaccharides (LPS) stimulated microglial cells at a maximal dose (100 μg/mL), and the extract significantly decreased the production of ROS, nitric oxide (NO), tumour necrosis factor-α (TNF-α), interleukin-1 (IL-1), and IL-6 (Table 1). The extract of *F. deltoidea* considerably decreased the ultraviolet (UV)-induced production of TNF-α, IL-1, IL-6, and COX-2. The *F. deltoidea* extract may block proinflammatory cytokines, making it a potent remedy for skin conditions (Table 2). Numerous compounds that inhibit the immune response have been identified in plants. The first large class of plant chemicals, phenolic compounds, are crucial to many plant functions. Depending on environmental conditions, phenolic chemicals can accumulate in various plant tissues and cells during ontogenesis. It has been shown through research that, phenolic chemicals, many of which are found in the cell walls, vacuoles, and nuclei of cells, have anti-inflammatory and anti-septic characteristics (Table 2). Action mechanisms because of exposure to inflammatory substances, cells secrete arachidonic acid and inflammatory mediators like cytokines, serotonin, histamine, prostaglandins, leukotrienes, and vascular permeability and leukocyte recruitment are increased (Figure 3).

**Table 2.** Anti-inflammatory activity of FD

**S/N**	**Methods**	**Solvent**	**Plant parts**	**Concentrations**	**Major findings**	**Reference**
1	3-(4,5-dimethylthiazol-2-yl)-2,5 diphenyl tetrazolium bromide (MTT)	Aqueous		0.1, 1, 10, and 100 μg/mL	The extract greatly reduced ROS, NO, TNF-α, IL-1, and IL-6 production in microglial cells stimulated by LPS at the maximum dose (100 μg/mL)	[43]
2	*In vivo*		Leaves		*F. deltoidea* preserved trabecular bone microarchitecture and decreased and increased osteoclast and osteoblast cell numbers, respectively, protecting ovariectomy-induced osteoporosis (OP) mice from alveolar bone loss	[44]
3	Lipoxygenase inhibition assay	Methanol	Leaves	10 μL	Using apigenin, nordihydroguaiaretic acid, and indomethacin as a control, the extracts’ activity was determined to be equivalent at *P* < 0.05	[11]
4	Enzyme-linked immunosorbent assay (ELISA)	Hot aqueous	Leaves	0.05, 0.08, or 0.1%	The UV-induced expression of TNF-α, IL-1, IL-6, and COX-2 was significantly reduced when the extract of *F. deltoidea* was used. Pro-inflammatory cytokines may be inhibited by the *F. deltoidea* extract, which may be an effective skin disease treatment	[45]
5	*In vivo*			200 and 400 mg/kg	The dose-dependent down-regulation of pro-inflammatory nuclear factor-kappa B (NF-κB), tumor necrosis factor alpha (TNF-α), and IL-6 mRNA levels by the FD extract considerably at *P* < 0.05 alleviated these bone microstructural and biomarker alterations. In this OP/osteoarthritis (OA) preclinical model, the FD extract showed good anti-osteoporotic characteristics by increasing bone formation and reducing bone resorption via anti-inflammatory pathways	[46]
6		Aqueous, ethanolic	Leaves		Biomarkers related to endothelial activation and inflammation were inhibited by FD at the highest levels, whereas FD reduced monocyte binding at the second-highest level (17.3%)	[47]
7	*In vivo*		Leaves	400 mg/kg	Radiological, macroscopic, and histological images revealed that osteoarthritic rats treated with the extract plus diclofenac had significantly less cartilage loss than osteoarthritic rats not treated with either substance. Osteoarthritic cartilage showed a substantial decrease in IL-1, prostaglandin E2 (PGE2) receptor, and matrix metalloproteinase-1 mRNA levels when the extract was applied	[48]
8	*In vivo*	Aqueous	Leaves	30, 100, and 300 mg/kg	The data demonstrated that FDA had a dose-dependent anti-inflammatory impact in the paw oedema and formalin tests and that it was anti-inflammatory in all assays tested (*P* < 0.05)	[42]
9	*In vivo*	Aqueous	Leaves	1, 50, and 100 mg/kg	Present results demonstrated that a dose-dependent antinociceptive effect was produced in all models by intraperitoneal administration of the *F. deltoidea* leaves aqueous extract 30 min before pain induction, indicating the presence of both centrally and peripherally mediated activities	[15]
10		Methanol			There was a dose-dependent inhibition of NO and proinflammatory cytokine production, including TNF-α, IL-6, and IL-1, by *F. deltoidea* ethyl acetate fraction compared to the other fractions. Acetate fraction treatment also reduced the expression of inducible NO synthase, NO synthase, and COX-2. Aside from these two effects, it also inhibited LPS-induced activation of NF-κB (an inhibitor of kappa B alpha) degradation	[49]

### Effects on microorganisms

**Figure 4.**
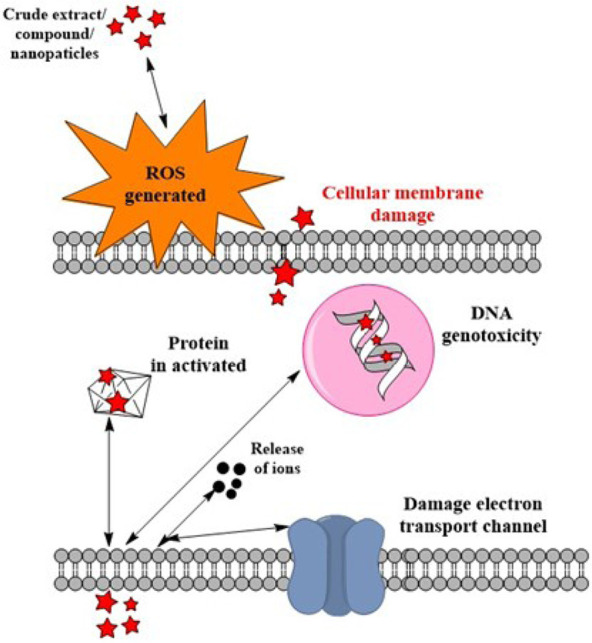
Mechanism of action of *F. deltoidea* against microorganisms. Pentagrams: compounds releases by the extract

By measuring the minimum inhibitory concentrations (MICs) and the diameter of the zone of inhibition, the antimicrobial activity against bacteria and fungi was tested (Table 3). The utility of *F. deltoidea* extracts against Gram-positive and Gram-negative bacteria are extensively known (Table 3). According to scientific research on *F. deltoidea*, these plants have garnered increasing interest in recent years. At a concentration of 31.26 mg/L, the plant extract did not stimulate the growth of the bacteria *Edwardsiella tarda*, *Escherichia* (*E.*) *coli*, *Flavobacterium sp.*, *Pseudomonas aeruginosa*, or *Vibrio cholera* (Table 3). An extract from the plant prevented the growth of *Aeromonas hydrophila*, *Klebsiella sp.*, *Salmonella sp.*, and *Vibrio alginolyticus* when administered at a dosage of 62.5 mg/L. At a concentration of 125 mg/L, the plant extract inhibits the expansion of the pathogen *Vibrio parahaemolyticus* [10]. A 10–12 mm inhibition was found against the tested bacterial strain [50]. All bacteria tested were inhibited by the extract; however, *Bacillus* (*B.*) *subtilis* showed the greatest inhibition at 12 mm [51]. When tested against *Staphylococcus* (*S.*) *aureus*, the plant extract exhibited an inhibitory zone of 15.67 mm and a MIC of 3.125 mg/mL. The smallest reported sensitivity to chloroform extract was 6.33 mm, while the largest MIC was 25 mg/mL, both for *B. subtilis* [52]. Except for chloroform and aqueous extracts of *B. subtilis*, *E. coli*, and *P. aeroginosa*, all extracts demonstrated inhibitory effects on the fungi, Gram-positive and Gram-negative bacteria [5]. Results showed that the methanol extract was effective against the bacteria and the fungi used in the tests. The methanol extract showed the lowest MIC value (3.125 mg/mL) and the widest inhibition zone (15.67 mm) against the growth of *S. aureus*. *B. subtilis* had the highest MIC value (25 mg/mL) and the lowest sensitivity (6.33 mm) to the chloroform extract [5]. At 50 and 100 mg/mL, the MIC and minimum fungicidal concentration (MFC) for *Candida albicans* were both achieved with the studied extracts. The extract had a 69.5% inhibitory effect on biofilm formation by Candida [53]. All test organisms showed that the extracts had a strong antimicrobial effect (Table 3). The qualitative and quantitative variability in the antifungal characteristics of the extracts is the root cause of the diversity in the inhibitory impact of plant extracts. The antimicrobial properties of these species may have come from the alkaloids, flavonoids, and cardiac glycosides found in these species’ leaves. Based on our findings, *F. deltoidea* extracts may be a viable alternative to antibiotics for managing drug-resistant bacterial and fungal strains. Apparently, the secondary metabolites in this plant are responsible for the extract’s extensive antibacterial activity. Various phytochemical substances have been reported to give *F. deltoidea* its medicinal benefits [1]. Species of the fig tree, *F.*, are excellent resources for polyphenolic chemicals. The enhanced efficacy of the extracts is considered due to the synergistic effects of the various bioactive compounds present in *F. deltoidea*. In particular, the released chemicals attach easily to the negatively charged cell wall and break it, causing protein denaturation and cell death in microorganisms (Figure 4), ultimately leading to its rupture and denaturation of its proteins and the death of the cell.

**Table 3.** Antimicrobial activity of FD

**S/N**	**Biological evaluation**	**Methods**	**Solvent**	**Plant parts**	**Concentrations**	**Major findings**	**Reference**
1	Antibacterial	Spread plate technique	Physiological saline	Rhizosphere	100 μL	Antibacterial tests showed that 61.8% of all isolates were wrought to *B. subtilis*, *S. aureus*, and *E. coli*, the tested bacteria	[54]
2		Agar dilution method	Petroleum ether chloroform methanol water	Leaves	1–128 mg/L and 1,024 mg/L	At 12 mm, the ethanolic extract inhibits *Helicobacter pylori* the most, while the aqueous extract has on activity	[55]
3		Disc	Hexane, ethyl acetate and methanol	Leaves		The MIC against *E. coli* is 230, and *B. subtilis* and *S. aureus* are 380 and 460 μg/mL, respectively	[56]
4		Disc	80% Ethanol			According to research, plants have a dynamic ability as pharmaco therapeutic agents	[57]
5		Disc diffusion	Chloroform, methanol and aqueous		0, 20, and 50 mg/mL	The methanol extract inhibited *S. aureus* growth significantly, forming a 15.7 mm wide inhibition zone with a minimum inhibitory dose of 3.125 mg/mL	[52]
6		Broth micro dilution, MIC, and minimum bactericidal concentration (MBC)	Distilled water	Leaves and stem oil	0.08–10 mg/mL	The oils inhibited all microorganisms tested moderately to strongly, with MIC and MBC values ranging from 0.63 mg/mL to 2.5 mg/mL	[58]
7		Micro dilution	Methanol, chloroform, ethyl acetate and butanol	Leaves	0.01–100 mg/mL	The leaves extract inhibited *S. aureus* more effectively than MRSA. Even though chloroform and methanol extracts of 100 mg/mL inhibited *S. aureus* by 20% and 16%, respectively, they did not affect MRSA	[1]
8		Disk diffusion and MIC	Hexane, ethyl acetate and methanol	Leaves (lupeol)		Lupeol is more sensitive to *S. aureus* than *E. coli* and more sensitive to *B. subtilis* than other antibiotics, according to the antibacterial activity test. The MICs of *E. coli*, *B. subtilis*, and *S. aureus*, respectively, were 150, 220, and 130 g/mL	[9]
9		MIC	70% Methanol	Leaves	31.26–125 mg/L	The plant extract failed to grow *Edwardsiella tarda*, *E. coli*, *Flavobacterium sp.*, *Pseudomonas aeruginosa*, and *Vibrio cholerae* at a dose of 31.26 mg/L. At a dosage of 62.5 mg/L, the plant extract inhibited *Aeromonas hydrophila*, *Klebsiella sp.*, *Salmonella sp.*, and *Vibrio alginolyticus*. The plant extract suppresses the growth of *Vibrio parahaemolyticus* at a dose of 125 mg/L	[10]
10		Disc	Ethanolic	Leaves		Inhibition of 10–12 mm was found against the tested bacterial strains	[50]
11			Methanol	Leaves	20 μL (10 mg/mL)	The extracts were found to inhibit all tested bacterial strains, with the highest inhibition against *B. subtilis* at 12 mm	[51]
12		Diffusion method	n-hexane, ethyl acetate, methanol	Leaves		The outcomes demonstrated the presence of nonpolar, semipolar, and polar antibacterial chemicals in the leaf extract. With a concentration of 15% and a clear zone diameter of 22.33 mm, *B. substilis* bacteria were the most harmed by the hexane extract. This level of inhibition is really strong	[59]
13		Diffusion method	Methanol	Leaves		The leaves’ methanolic extract inhibits the development of all investigated bacterial strains with MIC values of 1% for *S. aureus*, 0.7% for *S. epidermis*, 0.8% for *S. aeruginosa*, and 0.7% for *E. coli*	[60]
14	Antibacterial	Disc	Ethanol	Leaves		Diverse species of *F.* exhibited weak *in vitro* antibacterial activity against *Citrobacter* (*C.*) *freundii* isolated from locally infected *Anguilla anguil-la* L	[61]
15			Methanol and aqueous	Leaves and stems		Based on the results, leaf extracts of the two types could inhibit the development of all three bacteria, with a minimum inhibition concentration of 25 mg/mL. At a minimal inhibitory dose of 100 mg/mL, the stem aqueous extracts revealed inhibition zones against *P. aureginosa* and *S. aureus*. However, no inhibition zone was found in the stem methanol extracts	[62]
16	Antifungal	Disc	Aqueous	Leaves	5, 10, 15, and 20% (v/v)	The mycelia growth of *Ganoderma boninense* and *Rhizoctonia solani* was strongly reduced by extracts of *F. deltoidea* at all concentrations of more than 10%. *F. deltoidea* reveals the presence of one or more secondary metabolites that have antifungal properties	[63]
17		Disc	Physiological saline	Rhizosphere		The antifungal test revealed that 64.8% of the isolates were antifungal against four strains of *Fusarium oxysporum*, *Candida albicans*, and *Colletotrichum capsicus*	[54]
18		Disc	Chloroform, methanol and aqueous	Leaves	10, 20, and 50 mg/mL	All concentrations inhibited the growth of the tested strain	[52]
19		MIC and MFC		Leaves		Extracts tested positive for antifungal activity against *Candida albicans* at MIC and MFC levels of 50 and 100 mg/mL, respectively. The extract of 69.5% inhibited *Candida* biofilm development	[53]
20	Anti-plasmodia	Schizont maturation inhibition assay	Petroleum ether and ethanol	Leaves	1,000 μg/mL	Crude hydro-alcoholic leaf extracts have an IC_50_ of more than 50 μg/mL, whereas petroleum ether leaf extracts have a low IC_50_ of about 26 μg/mL	[64]

### Effects on the endocrine system

**Figure 5.**
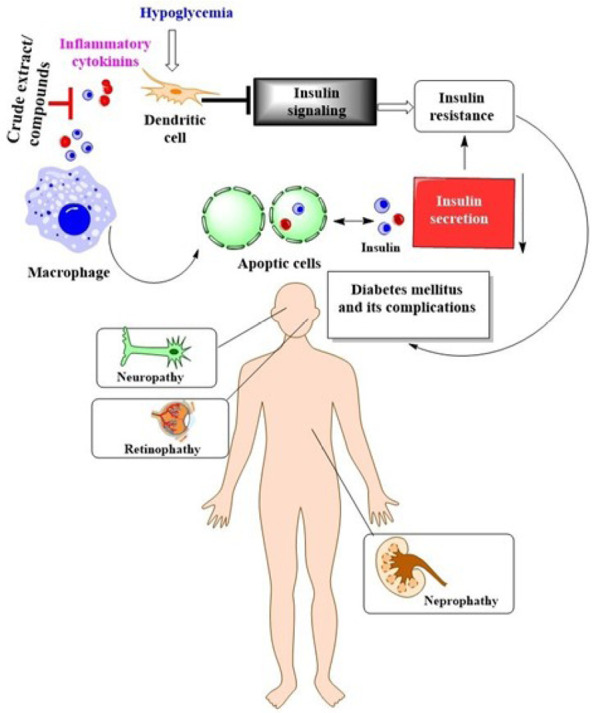
Mechanism of action of *F. deltoidea* against hypoglycemia

About 1.9 billion persons globally are overweight, and about 600 million are clinically obese [65]. This makes obesity the largest public health problem in the world today [65]. Fat accumulation in the cytoplasm of adipocytes defines the increased adipose cell size characteristic of obesity [66]. Several enzymes, including fatty acid synthase, lipoprotein lipase, and adipocyte fatty acid-binding protein, control this metabolic shift in adipocytes [66]. People have used traditional medicinal plants and their active phyto constituents to treat obesity and the problems that come with it. There is much-untapped potential in natural products for treating obesity, and they could be a great substitute for developing safe and effective anti-obesity medications. After therapy with *F. deltoidea* at 500 and 1,000 mg/kg/day, insulin resistance, obesity index, TC, triglycerides, low-density lipoprotein (LDL) cholesterol, MDA, testosterone, and follicle-stimulating hormone (FSH) were decreased to nearly normal levels in polycystic ovary syndrome (PCOS) rats (Table 4). The capacity of *F. deltoidea* leaf extracts to prevent the development of mature adipocytes suggests that they may have anti-obesity capabilities [67]. The findings showed that *F. deltoidea* is a viable medicinal plant for creating novel functional foods, herbal medicines, and contemporary drugs with enormous potential for treating obesity. It has been demonstrated through scientific research that *F. deltoidea* can lower hyperglycaemia in a variety of prandial situations (Table 4). Different studies have claimed that *F. deltoidea* has antidiabetic and antioxidant properties, but most of these investigations have only employed the leaf. Researchers have found a link between the phenolic content of plants and their ability to combat diabetes [18]. Increased protein content and lower glucosidase activity in treated *F. deltoidea* samples provide compelling evidence for the critical role of proteins in demonstrating the beneficial antidiabetic effect [18]. Some research has suggested that *F. deltoidea* antihyperglycemic effects are mediated by the plant’s ability to increase insulin secretion from pancreatic cells, boost adipocyte glucose uptake, and boost adiponectin release from adipocytes [68]. Particularly, flavonoids and isoflavonoids are because of the high levels of antioxidant activity found in the extract, which benefits in awarding against illnesses caused by oxidative damage [25]. The findings suggested that the extracts may be a viable antibiotic option for regulating the growth of various bacterial strains. Pure substances or crude extracts may work through the production of inflammatory cytokines, leading to the death of microphages in the circulation and the release of secretory insulin. On the other hand, stimulating dendritic cells in the brain, where Hypoglycemia is present, will indicate insulin release. By inhibiting the connection between the insulin receptor in the cells and insulin release, the presence of fat that covers the insulin receptor likely contributes to insulin resistance and diabetes mellitus. The condition depicted in Figure 5 highlights complications associated with diabetes mellitus: The neurological system is harmed by nephropathy, retinopathy, and neuropathy (damage to nephrons).

**Table 4.** FD effects on the endocrine system

**S/N**	**Methods**	**Solvent**	**Plant parts**	**Concentrations**	**Major findings**	**Reference**
1		Aqueous	Leaves		Leaf extracts of *F. deltoidea* may have anti-obesity properties based on their ability to inhibit the formation of mature adipocytes	[67]
2	*In vivo*	Methanol	Leaves	250, 500, or 1,000 mg/kg/day	Insulin resistance, obesity index, TC, triglycerides, LDL cholesterol, MDA, testosterone and FSH were reduced to near-normal levels in PCOS rats after treatment with *F. deltoidea* at 500 and 1000 mg/kg/day	[69]
3	Alpha-glycosidase and alpha-amylase assay	Methanol	Leaves	50 μL	The extract showed strong alpha-glycosidase and alpha-amylase inhibitory actions (IC_50_ values of 15.1 and 39.42 μg/mL, respectively)	
4	*In vivo*	Hot aqueous, ethanol, methanol		1,000 mg kgG^1^	The equivalent IC_50_ values for the hot aqueous, ethanolic and methanolic extracts are 4.15, 2.06, and 1.72 mg mLG^1^, respectively. All extracts suppressed alpha glycosidase activity through a mixed-type inhibitory mechanism, according to kinetic analyses of the enzymes	[70]
5	*In vivo*	Hot aqueous	Leaves		Leaves hot aqueous extracts significantly boosted insulin secretion in experiments, with a 7.31-fold increase in stimulation at *P* < 0.001	[68]
6	Glucose uptake assay	Hot and cold aqueous	Leaves		All the fractions except methanolic and n-Butanol possess insulin-mimetic activity	[71]
7	*In vivo*	Aqueous and n-hexane	Endophytic actinobacteria		Seventy-seven per cent of the 40 *F. deltoidea* endophytic actinobacteria isolates tested showed inhibitory action against rats (alpha-glucosidase)	[72]
8	Chang cells as the model of liver cells	Ethanol and methanol	Leaves	10–1,000 μg/mL	Researchers found that *F. deltoidea* extract significantly increased basal and insulin-mediated glucose absorption by 1.45 to 2.11-fold (*P* < 0.001). Insulin-mediated uptake was significantly more active than in the absence of insulin (*P* < 0.001)	[73]
9	Glucose uptake assay	Ethanolic, methanolic, and hot aqueous	Leaves	10–1,000 μg/mL	FD extracts significantly boosted basal or insulin-mediated glucose uptake in muscle cells at dosages. Aqueous extract at low concentrations (10 μg/mL) promoted glucose uptake, but the extract at high concentrations (500 and 1,000 μg/mL) promoted basal glucose uptake	[74]
10	*In vivo*		Leaves	350 mg twice daily	Fasting blood sugar, hemoglobin A1c (HbA1C) levels, renal function, or lipid profiles were unaffected. Patients in the intervention group claimed to feel more invigorated and revitalized than those in the control group	[75]
11		Methanol		10–1,000 μg/mL	Based on these findings, the modern candidate for antidiabetic drugs that target insulin secretion escalation from beta cells in the pancreas is the typical methanolic extract of FD variants	[76]
12	Glucose-responsive clonal insulin- secreting cell line	Aqueous	Leaves	10–1,000 μg/mL	FD extract at a dosage of 1,000 mg/mL significantly enhanced insulin secretion by 110%. In adipocyte 3T3F442A cells, glucose uptake was increased by an extra 41% at a concentration of 1,000 μg/mL in the baseline state and by an additional 35% at a concentration of 100 μg/mL in the insulin-mediated state	[77]
13	*In vivo*	50, 70, 80, 90, and 95% ethanol	Leaves	5 g/kg	These findings suggest that FD, by downregulating protein tyrosine phosphatase 1B (PTP1B), may improve insulin sensitivity, reduce hepatic glucose production, and increase glucose absorption in type 2 diabetes mellitus	[78]
14	α-Glycosidase inhibition assay	Hot aqueous	Leaves	156–5,000 μg/mL	A concentration of 1,000 μg/mL of *F. deltoidea* considerably increased insulin secretion from pancreatic P-cells, with an increase of 7.31-fold (*P* < 0.001)	[79]
15	*In vivo*	Methanol	Leaves		Both treatments significantly increased SOD, GPx, and thiobarbituric acid reactive substances (TBARS) activity. Additionally, the amount of TBARS reduced markedly	[80]
16	Yeast α-glucosidase inhibition assay	Hot aqueous		125 μL	There were dose-dependent inhibitory effects on the activity of yeast and rats in the gut but no effect on porcine pancreatic -amylase. In terms of α-glucosidase inhibition, the water fraction had the highest protein content at 73.33 μg/mg fraction	[18]
17	*In vivo*	Methanol, n-hexane, chloroform, n-butanol	Leaves	100, 200, and 400 mg/kg	Hydrophilic butanol sub-extract only showed blood glucose-lowering activity in normal mice, whereas methanol extract demonstrated blood glucose-lowering activity in both diabetic rats and normal mice. Methanol extract may contain insulin receptor sensitization and secretagogue components	[81]
18	*In vivo*		Leaves	1, 3, and 15 mg/kg	In normoglycemic mice given sucrose at 30 min, oral treatment of 1 mg/kg of vitexin (1) or isovitexin (2) significantly decreased postprandial blood glucose levels at *P* < 0.05. The percentage of postprandial blood glucose reduction was the highest with oral administration of 200 mg/kg isovitexin or 100 mg/kg isovitexin to sucrose-loaded diabetic rats	[82]
19	*In vivo*	Petroleum ether, chloroform, and methanol	Leaves	250, 500, and 1,000 mg/kg	After administering methanol extract by mouth, glucose tolerance improved. The antidiabetic effect of the methanol extract was highly significant at *P* < 0.01. In streptozotocin-induced diabetic rats, the extract therapy significantly reduced fasting blood glucose levels at *P* < 0.01. The extract treatment significantly halted weight loss in rats after streptozotocin administration	[83]
20	*In vivo*	Ethanolic aqueous	Leaves	250 and 500 mg/kg	*F. deltoidea* did not cause severe hypoglycemia when the extracts were given to normal rats at doses of 250 and 500 mg/kg. However, in the oral glucose tolerance test (OGTT), the leaf extracts reduced plasma glucose levels significantly after 30 min, albeit at varying levels, with *F. deltoidea* var. *intermedia* is the most efficient	[84]
21	*In vivo*	Hot aqueous	Leaves	100, 500, and 1,000 mg/kg	Two hours after giving 1,000 mg/kg of aqueous extract to a mildly diabetic rat, the rat’s blood sugar level dropped	[85]
22	*In vivo*			250 mg/kg	An elevated blood sugar level was reduced to an acceptable level following 30 days of treatment with *F. deltoidea* varietal *trengganuensis*, varietal *arteleri*, and varietal *intermedia* standardized extracts. In diabetic rats, the extracts substantially impacted the biochemical markers	[86]
23			Leaves		Vitexin and isovitexin, pungent components of *F. deltoidea* leaves, significantly inhibited amylase at *P* < 0.05 in an ethanol-water extract at 50%	[39]
24	*In vivo*	Ethanolic	Leaves	500 and 1,000 mg/kg body weight (BW)	Ethanolic extract of *F. deltoidea* reduced fasting blood glucose, particularly after 6 h of administration at all doses tested. When compared to metformin, the extract did not cause severe hypoglycemia. After 4 and 6 h, postprandial hyperglycaemia was significantly reduced as well	[87]
25	α-Glucosidase inhibitory assay	Methanol	Leaves	10 μL	The anti-diabetic results showed that var. *deltoidea*, var. *bornensis*, var. *intermedia*, var. *bilobata*, var. *kunstleri*, var. *trengganuensis* and var. *motleyana* displayed glucosidase inhibition with IC_50_ values of 6, 20, 26, and 36.5 μg/mL, respectively	[40]
26	*In vivo*	Aqueous	Fruits	150 and 300 mg/kg	The α-glucosidase assay revealed the highest protein concentration of 73.33 μg/mg in the aqueous fraction	[88]
27	*In vivo*	Aqueous	Leaves		Using aqueous extracts of all plants tested, blood glucose levels in rats were reduced by up to 50% over three to four weeks. Extraction from *Lagerstroemia speciosa*, followed by FD and *Areca catechu*, reduced blood glucose levels by the most, according to a new study	[89]
28	*In vivo*		Leaves		A combination of *F. deltoidea* and vitexin improved pancreatic antioxidant enzymes and boosted islet regeneration in a dose-dependent manner. In contrast, rats treated with *F. deltoidea* had significantly higher insulin secretion	[90]
29	*In vivo*	Methanol	Leaves	1,000 mg/kg BW	When *F. deltoidea* was given to diabetic rats, bone mineral density (BMD) went from 526.98 to 637.74, which is a big change. Compared to diabetic control, *F. deltoidea* treatment led to higher levels of insulin (2.41 *vs.* 1.58), osteocalcin (155.66 *vs.* 14.35), and total bone n-3 PUFA (2.34 *vs.* 1.44). Chondrocyte hypertrophy was also present	[91]

### Antihypertensive activity

There is a strong association between hypertension and the development of cardiovascular disease [92]. Over 1 billion people throughout the world are afflicted with it. Hypertension is more common in the elderly than in the young [92]. People over 60 have a prevalence of 65.5% of hypertension. Insulin resistance, obesity, glucose intolerance, concomitance, haemagglutinin, excessive uric acidemia, atherosclerosis, and cardiovascular illnesses are just a few of the chronic diseases and difficulties connected to hypertension [92].

High blood pressure in spontaneously hypertensive rats (SHR) can be reduced by administering an ethanol and water extract of FD. The renin-angiotensin-aldosterone system (RAAS) pathway, antioxidants, and the endothelial system may all play a role in this [93]. Rats treated with FD extract and losartan had significantly reduced blood pressure, heart rate, and heart weight compared to the controls (Table 5). The systolic blood pressure (SBP) of rats treated with FD plus losartan for four weeks was significantly lower than that of rats not treated with FD. Urine spectral analysis revealed 24 putative biomarkers with significance estimates greater than 0.5 (Table 1). Results from this study show that *F. deltoidea* has strong antihypertensive efficacy and shows promise for further research and development as an antihypertensive drug.

**Table 5.** Antihypertensive activity of FD

**S/N**	**Methods**	**Solvent**	**Concentrations**	**Major findings**	**Reference**
1	*In vivo*	Ethanol and aqueous	500, 800, 1,000, and 1,300 mg/kg	The ethanol and water extract of FDK lowers blood pressure in SHR. This could be due to RAAS, antioxidant, and endothelial systems	[93]
2	*In vivo*	Ethanol-water	800 or 1,000 mg/kg/day	When FDA and losartan were used to treat rats, the rats’ blood pressure, heart rate, and heart weight were all much lower than the controls	[94]
3	*In vivo*		1,000 mg/kg/day	After four weeks of treatment with FD, the SBP of rats treated with FD and losartan was much lower than that of rats that were not treated. An analysis of the spectra of urine showed that there were 24 possible biomarkers with variable importance projections above 0.5	[95]
4			1,000 mg/kg	Compared to the control group, rats treated with 1,000 mg/kg of FD and losartan demonstrated a significant decrease in blood pressure. Rats treated with FDK had decreased serum concentrations of angiotensin II and aldosterone compared to controls and rats treated with losartan. There were no variations between serum and urine electrolytes	[96]

### Aphrodisiac activity

Disturbances in a man’s sex drive might manifest as erectile dysfunction, ejaculatory dysfunction, or even an orgasmic condition [97]. Sexual activity is necessary for all humans and has been shown to increase the link between husband and wife, making it a good indicator of marital satisfaction. Psychological discomfort, infertility, and even suicide have all been linked to sexual dysfunction. Male mice benefit from an ethanolic extract from the leaves because it increases fertility and reproductive hormones (Table 1). Testosterone levels, sperm counts, and rat mobility improved after administering an aqueous and an ethanolic extract of FD leaves (Table 6). These treatments also substantially impacted the lowering of blood glucose levels, the number of abnormal sperm, and the frequency with which blood clots formed. Alloxan monohydrate’s negative effects on blood clotting, sperm quality, and testosterone level in male rats can be mitigated by administering an oral dose of an aqueous and ethanolic extract of FD leaves [98]. The sperm count, LDH-C 4 activity, and testosterone concentration of rats with diabetes were all enhanced after oral administration of an aqueous and ethanolic extract of FD leaves. These treatments had a major effect on blood glucose levels and sperm abnormalities [99]. Studies have demonstrated that plant polysaccharides help prevent testicular injury and encourage the renewal of testicular architecture. This study’s findings suggest that *F. deltoidea* extracts enhance general sexual performance and may also be useful in treating erectile dysfunction. The results support traditional medical claims that these plants have aphrodisiac properties and could improve sexual performance. These findings support the legitimacy of traditional Indian medicine’s recommendation for these plants.

**Table 6.** Aphrodisiac activity of FD

**S/N**	**Methods**	**Solvent**	**Plant parts**	**Concentrations**	**Major findings**	**Reference**
1	*In vivo*	Ethanol	Leaves	125, 250, 500, and 1,000 mg/kg BW for 28 days	By enhancing fertility, reproductive hormones, and antioxidant activity, the ethanolic extract of the leaves has a positive impact on the reproduction of male mice	[100]
2	*In vivo*	Hot aqueous	Leaves	0.125–4.0 mg/mL	The uterus of the isolated rat was subjected to a dose-dependent constriction by the FDA. As a result, maximum force of contraction (Emax) was lowered by all three of the drugs studied, with atosiban having a greater effect. The Emax was likewise lowered after oxodipine and ethylenediamine tetraacetic acid (EDTA) treatment. After the 2-aminoethoxydiphenyl borate (2-APB) administration, there was no noticeable difference. Thapsigargin, on the other hand, increased Emax	[101]
3	*In vivo*	Aqueous and ethanolic	Leaves	800 mg/kg	Oral administration of *F. deltoidea* leaf aqueous and ethanolic extract improved sperm count, LDH-C 4 activity, and testosterone concentration in diabetic rats. In addition to lowering blood glucose and sperm abnormalities, these treatments had a considerable impact	[99]
4	*In vivo*	Methanol	Fruits	50 mg/kg	Taking *F. deltoidea* dramatically improved male fertility	[102]
5	*In vivo*	Aqueous and ethanolic	Leaves		When rats were given an aqueous, and an ethanolic extract of *F. deltoidea* leaves, their testosterone level, sperm count, and mobility improved. Moreover, these treatments greatly reduced the blood glucose levels, the number of abnormal sperm, and the rate at which the blood clots. In conclusion, giving male rats an oral dose of the aqueous and ethanolic extract of *F. deltoidea* leaves may reverse the effects of alloxan monohydrate on blood clotting, sperm quality, and testosterone levels	[98]

### Wound healing activity

Major causes of physical incapacity include wounds [103]. A wound is a tissue disturbance brought on by physical, chemical, microbial, or functional losses [103]. Numerous factors, including bacterial infection, necrotic tissue, obstruction of the blood supply, lymphatic blockage, and diabetes mellitus, cause the wound healing process to be delayed (or reduced). Generally speaking, if any agent could change the aforementioned factors, the healing rate would be increased [104]. In Ayurveda, many plants play a crucial part in the recovery from injury. Plants are superior medicines because they work by stimulating the body’s natural mending processes [105]. Healing time is reduced, and aesthetics are preserved with plant-based therapy [106]. Animal products comprise less than 10% of wound-healing pharmaceuticals, whereas plants account for over 70%. Antiseptic coagulants and wound washes made from plant-based ingredients are utilized in emergencies [106]. Compared to wounds treated with sterile deionized water or dressed with a blank placebo, wounds treated with a placebo containing 5%, 10% *F. deltoidea* extract, or intrasite gel dramatically accelerated the healing rate [107]. A dose-dependent increase in cell proliferation can be achieved with leaf extract. In scratch testing, *F. deltoidea* leaf extract sped up wound healing compared to ascorbic acid-treated and untreated cells [108]. The 20% methanolic extract of leaves has been shown to speed up the healing process of wounds (Table 7). In terms of DNA and hydroxyl proline content, mice given an extract concentration of 80% showed the highest levels (Table 7). The extract’s wound-healing efficacy is proportional to its concentration (Table 7). FD extract is an effective treatment for wound healing since it can activate the body’s natural repair processes. The mechanism of *F. deltoidea* extract’s activity on the healing of wounds still must be understood.

**Table 7.** Wound healing activity of FD

**S/N**	**Methods**	**Solvent**	**Plant parts**	**Concentration**	**Major findings**	**Reference**
1	*In vivo*	Methanol	Leaves	20, 40, 60, and 80%	Methanolic extract of leaves at 20% concentration can heal wounds. The mice administered with an extract concentration of 80% exhibited the highest quantities of DNA and hydroxyl proline. The concentration of the extract influences how well it heals wounds	[109]
2	Scratch assays	Hot aqueous	Leaves		Leaf extract can stimulate cell growth in a dose-dependent way. Compared to cells treated with ascorbic acid and untreated cells, *F. deltoidea* leaf extract accelerated wound closure in scratch assays	[108]
3	*In vivo*	Aqueous	Whole plants	Placebo containing 5% and 10%	Wounds treated with *F. deltoidea* extract containing 5 or 10% of the total extract considerably expedited the healing process compared to wounds treated with sterile deionized water	[107]
4		Aqueous, methanol, and ethanol	Leaves	10–1,000 μg/mL	Inhibition of human liver glucuronidation activity was found in the range from 34.69 μg/mL to 398.10 μg/mL	[110]
5	*In vivo*	Hot aqueous	Leaves		The liver and kidneys were unaffected by the extract. Rats treated with the extract gained weight, improved depressed behaviour, and had fewer pyknotic and dark-stained neurons in their hippocampus	[111]

### Anticancer activity

**Figure 6.**
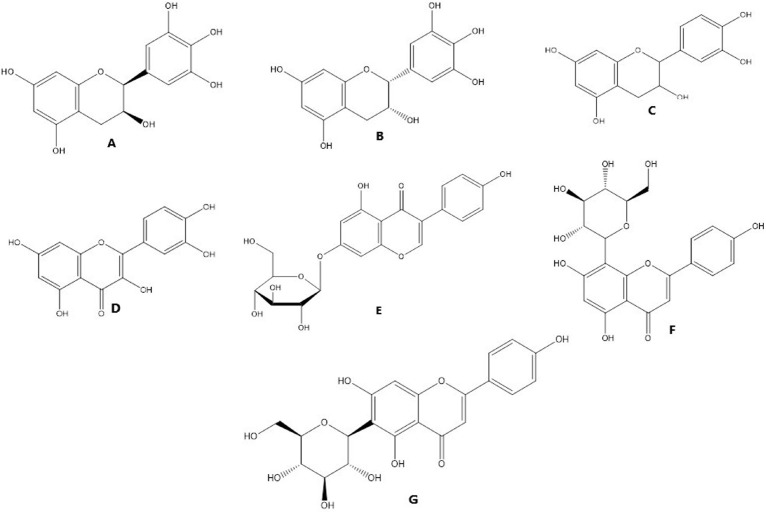
Some compounds responsible for the *F. deltoidea* biological activity. A. Gallocatechin; B. epigallocatechin; C. catechin; D. quercetin; E. Genistin; F. vitexin; G. isovitexin

**Figure 7.**
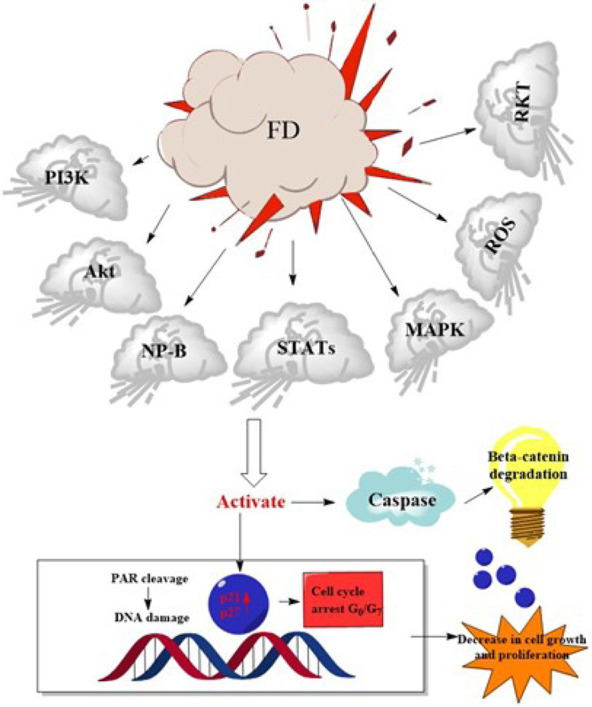
Possible mechanisms of action of *F. deltoidea* crude extract or pure compounds on cancer cells. STATs: Signal transducer and activator of transcription; RKT: Receptor tyrosine kinases; PAR: Protease-activated receptor; G_0_: Resting state; G_7_: Gap phase

There has been much focus on plant-based biological products for quite some time. The potential for finding novel biomolecules with future applications motivates the investigation of these priceless by-products. Natural plant products are becoming increasingly popular for use in both the prevention and treatment of disease. The traditional use of medicinal plants as a treatment method is the basis for contemporary medicine [112]. The success rate of treating cancer with allopathic medicine or chemotherapy drugs like cisplatin has increased over time [16]. However, this course of treatment is commonly cited as having dangerous side effects because of the toxicity of chemotherapeutic drugs. Additionally, chemoresistance is to blame for 90% of drug failures in patients with metastatic cancer [16]. Researchers have tried to find alternative treatment approaches to treat cancer, some of which involve using natural products. Drugs used in chemotherapy to treat cancer are typically based on chemicals first identified in plants or synthetic versions of these molecules [112]. Unfortunately, despite many efforts, cancer is still a major cause of death worldwide. Because of this, researchers are constantly looking for new, cost-effective treatments for cancer. Growing evidence suggests that compounds produced from plants may be able to inhibit several steps in carcinogenic and inflammation-related processes, making these products increasingly important in cancer prevention and treatment. Both 48.2 and 62.7 g/mL had IC_50_ values that suppressed microvascular proliferation (Table 8). Mice infected with azoxymethane/dextran sodium sulfate (AOM/DSS) had lower levels of alpha-catenin in their colons, which was inhibited by the FD ethanol extract. Human colon cancer (HCT 116) was also inhibited by the FD ethanol extract [113]. Nuclear DNA fragmentation showed that the extracts produced apoptosis, a form of cell death (*P* < 0.05). In PC3 and L ymph N ode Ca rcinoma of the P rostate (LNCaP) cell lines, there was also a substantial increase in mitochondrial membrane potential (MMP) depolarization (*P* < 0.05) and caspase 3 and 7 activations (*P* < 0.05) (Table 8). The IC_50_ values were calculated to be 224.39 μg/mL for the aqueous extract and 143.03 g/mL for the ethanolic extract. However, only the ethanolic extract (1,000 μg/mL) caused DNA fragmentation, while the water-based extract had no effect. The breaking caused a loss of about 200 kbp of DNA. Morphological testing revealed apoptotic bodies appeared in both extracts at concentrations of 1,000 μg/mL [114]. When tested for cytotoxic effects on the HL-60 cell line, it was discovered that the FD leaf extract was far more potent than the fruit extract [115]. The FD extract positively impacted tumour development. When the FD extract was used, the incidence of oral squamous cell cancer (OSCC) decreased from 100% to 14.3% in the high-dose groups [116]. At the end of the treatment period, there was a significant decrease in testosterone, FSH, and luteinizing hormone (LH) levels at *P* < 0.05 but a significant increase in progesterone and estrogen levels at *P* < 0.05 in extract treated groups compared to the control group [117]. We found that *F. deltoidea* extract significantly slowed the growth of established tumours, indicating that it possessed potent anticancer properties (Table 8). Flavonoids abundant in *F. deltoidea* include isovitexin, gallocatechin, ellagic acid, coumaroylquinic acid, catechin, gallic acid, quercetin (Figure 6), and naringenin. The anticancer benefits of the plant are due to these compounds [8]. *F. deltoidea* has high levels of polyphenolics, flavonoids (such as genistin), alkaloids (such as antofine), and tannins [118, 119]. Flavonoids like epigallocatechin have been proven to inhibit the growth of prostate cancer cells *in vitro* [120]. Vitexin has a cytotoxic effect on breast, ovary, and prostate cancer cells by upregulating *BCL2*-associated X protein (Bax) and downregulating BCL2 and causing the breakage of the poly[adenosine diphosphate (ADP)-ribose] polymerase (PARP) protein [121]. By decreasing the *BCL2*/Bax ratio and activating caspases, vitexin inhibits tumour growth and spread by killing cancer cells [122]. Naturally occurring antioxidant ellagic acid has been demonstrated to have antiproliferative and pro differentiation actions on prostate cancer cells via suppressing eicosanoid synthesis and the heme oxygenase system [123]. Murine leukaemia cells and the human lung cancer cell line have both been shown to undergo apoptosis when treated with the antioxidants rutin and quercetin, respectively, which have been linked to having anticancer characteristics. Plant polyphenols have long been recognized for their antioxidative effects against oxidative stress, which has been associated with cancer [119]. The flavonoids in *F. deltoidea* have therapeutic potential as a treatment for prostate cancer [122]. The possible mechanism of action of *F. deltoidea* as a tumour suppressor and its crude extract or pure components is shown in Figure 7. It also depicts the anti-tumorigenic activities induced by signal transducing components by crude extracts or pure chemicals and the tumour cascade pathways initiated in cancerous cells by various growth factors in Figure 7. The expression of the tumour-inducing pathways phosphoinositide 3-kinase (PI3K), protein kinase B (Akt), natriuretic peptide type B (NP-B), mitogen-activated protein kinase (MAPK), and ROS is downregulated at the infection site by crude extract or pure chemical in a conjugated form. Pure substance or crude extract interrupts the cycle, prevents the synthesis of p21 and p27 cyclin-dependent kinase inhibitors, and prevents the mitotic effects. These activities are all connected to cancer cell development, dissemination, and proliferation. *F. deltoidea* killed multiple tumour cell lines; however, the effectiveness was dose- and time-dependent. This analysis verified the results of ethnobotanical studies that reveal the medicinal potential of *F. deltoidea* used in traditional medicine. Based on our findings, *F. deltoidea* extracts or pure compounds have great potential as an anticancer drug.

**Table 8.** Anticancer activity of FD

**S/N**	**Methods**	**Solvent**	**Plant parts**	**Concentration**	**Major findings**	**Reference**
1	*In vivo*		Leaves	250 and 500 mg/kg	Administration of *F. deltoidea* leaf extract considerably decreased the size of the oral ulcer. Compared to the 250 mg/kg extract, the 500 mg/kg extract showed a larger proportion of the inhibitory oral ulcer area. According to the study’s findings, *F. deltoidea* extract can hasten the healing of oral ulcers, making it a potential therapeutic agent	[124]
2		Hot aqueous	Leaves	0.125 mg/mL	Treatment with *F. deltoidea* leaf extract effectively inhibited alpha-melanocyte stimulating hormone (MSH)-induced melanin formation in a dose-dependent manner comparable to that of kojic acid. The extract decreased mushroom tyrosinase activity as well as B16F1 intracellular tyrosinase activity	[125]
3	*In vivo*	Ethanolic	Leaves	25, 125, and 250 mg/kg BW	After the treatment period, a substantial decrease at *P* < 0.05 in testosterone, FSH, and LH levels was found, but a significant increase at *P* < 0.05 in progesterone and estrogen levels was found in extract-treated groups compared to the control group	[117]
4	*In vivo*				FD extract significantly reduced the incidence of OSCC in the high-dose group from 100% to 14.3%. Cellular adhesion-enhancing antibodies, such as β-catenin and E-cadherin antibodies were found to have been dramatically downregulated in tumours treated with the FD extract, according to immunohistochemistry examination	[16]
5	MTT	Hot aqueous	Leaves		As shown by the appearance of apoptotic bodies, fragmentation, cell blebbing, and shrinkage, the crude and its active fraction caused cell decrease through apoptotic machinery	[122]
6	*In vitro* and *in vivo*	Ethanol	Leaves	12.5%, 25%, and 50% w/v	Mice infected with AOM/DSS had lower levels of alpha-catenin in their colons, which was inhibited by the FD ethanol extract. The extract also inhibited HCT 116 with an IC_50_ value of 5.41 mg/mL	[113]
7	*In vivo*	Methanol and aqueous	Leaves		Extracts of methanol and water inhibited microvessel outgrowth with IC_50_ values of 48.2 and 62.7 g/mL, respectively	[126]
8	MTT	Hexane, ethyl acetate, methanol, and water	Leaves	0–500 μg/mL	The ethyl acetate extracts exhibited antiproliferative properties in breast cancer cell lines (MCF-7, MDA-MB 231), and human colorectal carcinoma cell line (HCT 116) cells with an IC_50_ value of 100 μg/mL and moderate anti-proliferative properties in hepatocellular carcinoma 1937 (HCC 1937) cells with IC_50_ values of 150–200 μg/mL	[12]
9	MTT	70% Methanol	Leaves		No anticancer activity was observed	[10]
10	MTT	Aqueous	Leaves	1–100 μg/mL	The cancer cell line was the most sensitive to the extract, with an IC_50_ value of 93.11 μg/mL. Therefore, the results suggested that there might be a link between antioxidant activity and bioactive markers in the prostate cancer cell line (DU145)	[30]
11		Methanol		30 μg/mL	Nuclear DNA fragmentation indicated that the extracts caused cell death through apoptosis (*P* < 0.05). There was also a significant uptick in MMP depolarization (*P* < 0.05) and caspases 3 and 7 activations (*P* < 0.05) in PC3 and LNCaP cell lines	[8]
12	MTT and trypan blue exclusion	Aqueous		1,000, 100, 10, 1, 0.1, 0.001, 0.0001, 0.00001, 0.000001, and 0.0000001 μg/mL	Human prostate cancer cells and normal fibroblast cells are killed at 1 × 103 (μg/mL) dose. The extracts altered the cells’ morphology; they were uneven, disconnected, and floated aimlessly in the liquid	[127]
13	MTT	Aqueous, and ethanolic		0–1,000 μg/mL	The IC_50_ was determined to be 224.39 μg/mL in aqueous extract and 143.03 μg/mL in ethanolic extract, respectively. The DNA fragmentation was only seen in the ethanolic extract (1,000 μg/mL) but not in the water-based extract, and about 200 kbp of DNA was lost in the shattering. The morphological analysis showed that apoptotic bodies were present at 1,000 μg/mL of both extracts	[114]
14	MTT	Methanol	Leaves and fruits	50 mg/kg BW/day	The FD leaf extract was shown to be more effective than the fruit extract in its cytotoxic activity against the HL-60 cell line	[115]

### Toxicity evaluation

Despite their efficacy in treating specific diseases, the widespread use of some medicinal plant species is associated with serious adverse effects. Many pharmaceuticals owe their existence to the discovery of a chemical in a plant that has biological activity and has subsequently been used to treat medical conditions. The natural chemical compounds in plants give them pharmacological and therapeutic properties, and their potential toxicity must be evaluated to ensure that the product is safe for human consumption (Table 9). Compared to the control group, no appreciable variations in the number of micro-nucleated cells were seen. At concentrations up to 5,000 g/plate, the extract was not found to increase the number of revertant colonies in any strains tested. In conclusion, more studies using animal models are necessary to confirm non-geno-harmful effects [128]. Uterine abnormalities in bisphenol A (BPA)-exposed rats improved significantly after six weeks of concurrent therapy with *F. deltoidea*. The histology of the myometrium and glandular epithelium appeared normal, and mitotic patterns were visible in the interstitial gaps between the stromal cells [129]. In an acute toxicity assay, the extract’s median lethal dose (LD_50_) was greater than at the concentration of 5,000 mg/kg. The sub-chronic toxicity study findings were shown to have no impact on food consumption, BW, organ weight, mortality, clinical chemistry, haematological, gross pathology, or histology (Table 1). All extracts had a higher than 2,000 mg/kg BW and LD_50_, and the acute toxicity test showed no signs of morbidity or mortality. Histopathological analyses of the kidneys and liver showed no abnormalities [84] despite the non-reportage of any toxic part of *F. deltoidea*. Additional testing is required using various cell lines in a range of sample dilutions. The medication is then tested in rodents and other animals, particularly mice and rats, before being administered to human patients.

**Table 9.** Toxicity evaluation of FD

**S/N**	**Methods**	**Solvent**	**Plant parts**	**Concentrations**	**Major findings**	**Reference**
1		Methanol, chloroform, ethyl acetate, and butanol	Leaves	0.01–100 mg/mL	The extracts also had an anti-proliferative activity that was dosage dependant. All three cell lines tested were practically ineffective against both butanol and ethyl acetate extracts (IC_50_ values > 1,000 g/mL). All three cell lines responded well after 48 h of treatment with methanol extract	[1]
2	Alkaline comet assay	Aqueous	Leaves	5, 2.5, 1.25, 0.625, 0.3125, and 0.15625 mg/mL	No significant differences were identified in the number of micro-nucleated cells compared to the control group. The extract did not enhance the number of revertant colonies in any strains tested at levels up to 5,000 μg/plate. To summarize, additional research in animal models is required to verify FDA’s non-geno toxic actions	[128]
3	*In vivo*	Methanol, n-hexane, chloroform, and n-butanol	Leaves	100, 200, and 400 mg/kg	Unlike hazardous chloroform and hexane sub extracts, hydrophilic methanol extract resulted in zero per cent mortality up to 6,400 mg/kg in 14 days. After four weeks of administration of 200 mg/kg, it did not generate liver or renal damage. The methanol extract revealed a low level of oral toxicity and diverse antidiabetic effects	[81]
4	MTT	Aqueous	Leaves and fruits	1 ng/mL to 1 mg/mL	Lowering the *F. deltoidea* leaf extract concentration from 1 mg/mL to 1 ng/mL increased cell viability	[21]
5	MTT	Aqueous		0.1, 1, 10, and 100 μg/mL	The extract was not harmful at any concentrations tested because microglial cell viability was consistently more than 80%	[43]
6	*In vivo*		Leaves	2,000 mg/kg	At oral doses of 2 g/kg, neither vitexin (1) nor isovitexin (2) showed any symptoms of toxicity in normoglycemic mice or diabetic rats	[82]
7	*In vivo*	Petroleum ether, chloroform, and methanol	Leaves	50–5,000 mg/kg BW	All the doses examined resulted in no treatment-related deaths. After 14 days, there were no significant changes in the animals’ behaviour, such as apathy and hyperactivity, as well as illness and mortality	[83]
8	MTT	Hot aqueous	Leaves		The maximum extract concentration that did not affect cell viability was 0.1% (w/v)	[125]
9			Leaves	1,000 mg/BW	This group did not affect glycaemia variables, although total and LDL cholesterol values were dramatically reduced. Vital signs and safety lab tests were within normal ranges at baseline and after 8 weeks of intervention, there were no significant differences between groups or attributable to the intervention	[130]
10	Brine shrimp lethality assay and *in vivo*	Aqueous	Leaves		According to the research, the extracts had no harmful effects on brine shrimp (up to 4,000 μg/mL) or rats (up to 0.2 per cent BW)	[89]
11	*In vivo*	Ethanolic	Leaves		The LD_50_ of the extract was found to be more than 5,000 mg/kg in an acute toxicity assay. Food consumption, BW, organ weight, mortality, clinical chemistry, haematological, gross pathology, and histopathology were all unaffected by the sub-chronic toxicity study results	[131]
12	*In vivo*		Leaves	1,000 mg/kg	It was shown that up to 1,000 mg/kg of *F. deltoidea* leaf extract was not harmful	[124]
13	*In vivo*	Aqueous	Leaves	100 mg/kg/day	Uterine abnormalities in the BPA-exposed rats significantly improved after six weeks of concomitant therapy with *F. deltoidea*. The myometrium and glandular epithelium histology seemed normal, and mitotic patterns were present in the interstitial gaps between the stromal cells	[129]
14	*In vivo*	Ethanol	Leaves	125, 250, 500, and 1,000 mg/kg BW for 28 days	The leaves’ ethanolic extract has no harmful effects and does not alter the histological structure of the testes	[100]
15	*In vivo*	Aqueous	Leaves	100 mg/kg/BW	The data demonstrated that *F. deltoidea* had a protective effect against BPA-induced ovarian damage. Normalization of FSH and sexual steroid hormone (progesterone) levels supported this conclusion	[132]
16	*In vivo*	Ethanolic aqueous	Leaves	5, 50, 300, and 2,000 mg/kg	The LD_50_ of the extracts for all kinds was higher than 2,000 mg/kg BW, and the acute toxicity test revealed no symptoms of morbidity or mortality. The kidneys and liver’s histopathological evaluation revealed no abnormalities	[84]
17	*In vivo*		Fruits		According to the testing data, the tensile strength of carbon nanotube (CNT)-filled composites increased by 7.73% compared to the control unfilled hybrid composites. For the CNT-filled composites, the flexural characteristics decreased by 49.37% compared to the control, which had no CNTs	[133]
18	*In vivo*	Methanol: distilled water (60:40 % v/v)	Leaves	300, 2,000, and 4,000 mg/kg	Some important organs underwent haematological and histological examination. Mortality was not recorded at any point during the study in either the acute or sub chronic toxicity groups Encapsulated plant extracts (600 and 1,000 mg/kg) increased serum glutamic oxaloacetic transaminase (SGOT) and serum glutamic pyruvic transaminase (SGPT) levels significantly, and histological assessment of the liver, kidneys, and spleen showed normal tissue limits	[134]
19	Viability assay	Methanol		100 μL	Viability was only shown to be hazardous at 500 and 1,000 μg/mL (*P* < 0.001)	[76]
20	*In vivo*	Ethanol	Leaves	125, 250, 500, and 1,000 mg/kg BW	The absence of toxic symptoms and death at a 2,000 mg/kg BW dose suggests that the LD_50_ was higher. Throughout this time, no changes in the mouse’s behaviour, substantial weight changes, haematological parameters, or serum biochemistry were noticed	[135]

## Discussion

The therapeutic properties of FD have been recognized for centuries, and the elderly have found several uses. Scientific research was conducted to confirm its effects, particularly in pharmaceutical applications, as it gained increasing attention. Its biological efficacy was documented in the current study. These findings provide solid evidence for the considerable and positive benefits of *F. deltoidea* extract on the rate of wound healing, cancer, fever, diabetes, blood pressure, bacterial infection, fungal infection, and many other diseases due to the presence of phenolic and flavonoid bioactive compounds. However, additional research into the bioactive components that may be responsible for its anticancer activity is necessary. Further studies must determine the appropriate dosage for treating and controlling cancer and related disorders globally. This study may serve as a solid foundation for creating herbal medicines or active compounds with tremendous potential for use in the treatment and prevention of cancer and its related future.

## Abbreviations

*B.*: *Bacillus*
BPA: bisphenol A
BW: body weight
CAT: catalase
CNT: carbon nanotube
COX: cyclooxygenase
DPPH: 2,2-diphenyl-1-picrylhydrazyl
*E.*: *Escherichia*
Emax: maximum force of contraction
*F.*: *Ficus*
FD: *Ficus deltoidea* Jack
FDA: *Ficus deltoidea* Jack aqueous extract
FRAP: ferric reducing antioxidant power
FSH: follicle-stimulating hormone
GPx: glutathione peroxidase
IC_50_: half maximal inhibitory concentration
IL-1: interleukin-1
LD_50_: median lethal dose
LDL: low-density lipoprotein
LPS: lipopolysaccharides
MFC: minimum fungicidal concentration
MICs: minimum inhibitory concentrations
MTT: 3-(4,5-dimethylthiazol-2-yl)-2,5 diphenyl tetrazolium bromide
NO: nitric oxide
ROS: reactive oxygen species
*S.*: *Staphylococcus*
SOD: superoxide dismutase
TC: total cholesterol
TNF-α: tumour necrosis factor-α

## Declarations

### Copyright

© The Author(s) 2023.
